# Progesterone resistance in endometriosis: A pathophysiological perspective and potential treatment alternatives

**DOI:** 10.1002/rmb2.12588

**Published:** 2024-06-07

**Authors:** Hsiao‐Chien Tang, Ting‐Chien Lin, Meng‐Hsing Wu, Shaw‐Jenq Tsai

**Affiliations:** ^1^ Institute of Basic Medical Sciences College of Medicine, National Cheng Kung University Tainan Taiwan; ^2^ Department of Gynecology and Obstetrics National Cheng Kung University Hospital Tainan Taiwan; ^3^ Department of Physiology College of Medicine, National Cheng Kung University Tainan Taiwan; ^4^ Department of Biomedical Sciences College of Science, National Chung Cheng University Chiayi Taiwan

**Keywords:** cell signaling, endometriosis, hormonal therapy, microRNA, progesterone resistance

## Abstract

**Background:**

Endometriosis is a common gynecological disease affecting women of reproductive age. Patients with endometriosis frequently experience severe chronic pain and have higher chances to experience infertility. Progesterone resistance is a major problem that develops during the medical treatment of endometriosis, which often leads to treatment failure of hormonal therapies. Previous studies indicated that the dysregulation of progesterone receptors (PR) is the primary factor leading to progesterone resistance in endometriosis.

**Methods:**

This review article systematically reviewed and summarized findings extracted from previously published papers available on PubMed, encompassing both experimental studies and clinical trials.

**Main findings:**

Various determinants influencing PR expression in endometriosis have been identified, including the environmental toxins, microRNAs, cell signaling pathways, genetic mutations, and the pro‐inflammatory cytokines. The selective estrogen/progesterone receptor modulators have emerged as novel therapeutic approaches for treating endometriosis, offering potential improvements in overcoming progesterone resistance.

**Conclusion:**

Concerns and limitations persist despite the newly developed drugs. Therefore, studies on unraveling new therapeutic targets based on the molecular mechanisms of progesterone resistance is warranted for the development potential alternatives to overcome hormonal treatment failure in endometriosis.

## 1 INTRODUCTION

### 1.1 Epidemiology of endometriosis

Endometriosis is a prevalent gynecological disease, which is characterized as a chronic inflammatory disease with the notable feature of endometrial tissues growing outside the uterine cavity. Endometriosis affects approximately 10% of women in their reproductive years, which is about 190 million women around the world. However, estimating the true endometriosis prevalence rate remains challenging because of the diagnosis requiring invasive approaches for observation and confirmation. In addition to the hidden population resulting from not actively seeking for clinical assistance or diagnostic delay. Based on the data analysis of global study of women's health, the diagnostic delay of endometriosis was 6.7 years, which primarily attributes to the delays of specialist referral.^1^ These results indicated that the real prevalent rate of endometriosis may be even higher than 10%.

**FIGURE 1:**
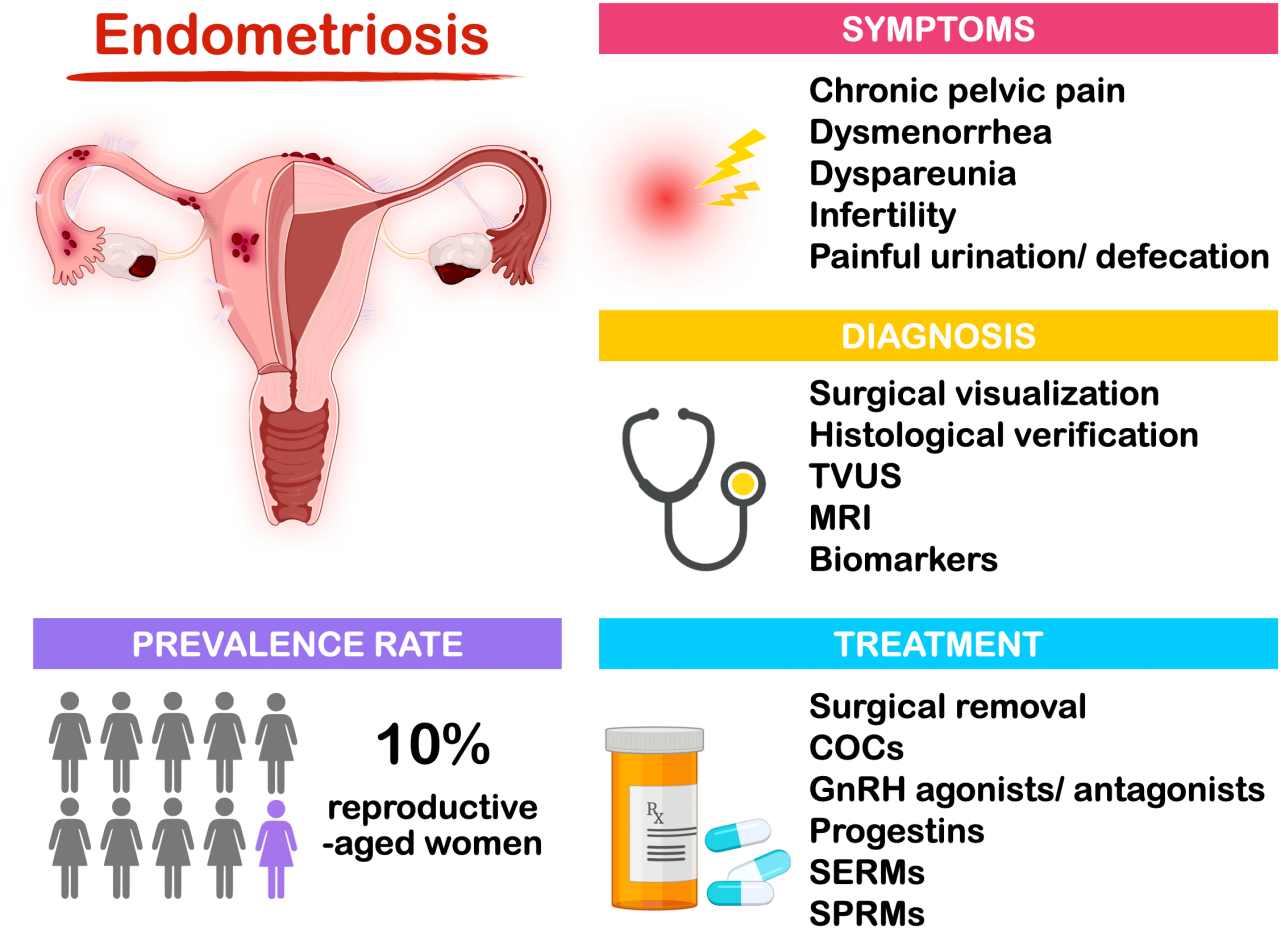
**Epidemiology and treatment of endometriosis.** According to WHO announcement in 2023, the prevalence rate of endometriosis is about 10% of reproductive‐aged women worldwide. Endometriosis patients often suffer from many different pain symptoms such as the chronic pelvic pain, dysmenorrhea, dyspareunia, and painful urination and defecation. However, the most serious problem for endometriosis patients is infertility. Nowadays, there are several diagnostic approaches ranging from traditional surgical method to application of imaging‐based devises and examination of biomarkers. The current treatments for endometriosis, including surgical removal and hormonal treatments. As for the novel therapeutic treatment, SERMs and SPRMs are the newly identified drugs, which have been reported to overcome the progesterone resistance in endometriosis. (COCs, combined oral contraceptive pills; MRI, magnetic resonance imaging; SERMs, selective estrogen receptor modulators; SPRMs, selective progesterone receptor modulators; TVUS, transvaginal ultrasonography).

Endometriosis patients usually suffer from chronic pelvic pain, dysmenorrhea, dyspareunia, infertility, and even have hard time on urination and defecation, which seriously reduces their daily life quality (Figure 1). A global scale study with a total of 1418 premenopausal women in 2011 revealed that the overall work productivity loss caused by endometriosis was significantly increased to 10.8 h/week comparing to the asymptomatic control.^1^ Recently, it was reported that the annual economic cost of Eastern Mediterranean endometriosis cases was estimated at Int$9864 (95% CI: $8811–$10 917), a figure contributed from the healthcare and the costs associated with the absence and loss of productivity at work.^2^ These results emphasize the serious inconvenience and financial burden accompanied with endometriosis.

### 1.2 Diagnosis of endometriosis

Surgical visualization with histological verification is the most reliable diagnostic approach; however, the advancement of new technology opens the door for less‐ or noninvasive diagnostic methods in detecting endometriosis (Figure 1). For example, it has been reported that ovarian endometrioma and deep infiltrating endometriosis (DE) can be detected through imaging‐based devices like transvaginal ultrasonography and magnetic resonance imaging.^3^ On the other hand, many studies have suggested that the higher levels of plasma molecules, including CA‐125, interleukin‐1β (IL‐1β), and vascular endothelial growth factor‐C (VEGF‐C), may be the potential endometriosis markers for noninvasive diagnostic approaches.^4^, ^5^, ^6^ Newly published research also demonstrated that the combining detection of CA‐125 and annexin A5 in serum appears to provide a more reliable diagnostic evaluation for moderate to severe (stage III and IV) endometriosis patients.^7^ Moreover, a preliminary report of gas chromatography–mass spectrometry study revealed that there were significantly lower fucose and N‐acetylglucosamine contents in serum immunoglobulin‐G from women with endometriosis compared to those without endometriosis, which gives a new concept for non‐invasive diagnostic parameters or algorithms.^8^ Nevertheless, the pursuit of noninvasive diagnostic markers with high specificity and sensitivity for early detection of endometriosis is indispensable, which enables the early therapeutic treatment and reduces the complications related to the disease.

### 1.3 Etiology and types of endometriosis

Several pathogenic theories of endometriosis have been proposed to date, including the metaplasia theory, stem/progenitor cell theory, lymphatic spread theory, and Müllerian rests theory.^9^, ^10^, ^11^, ^12^ The most widely accepted theory was proposed by Sampson, who suggests that endometriosis is originated from retrograded menstruation.^13^ According to Sampson's theory, the menstrual flow travels backward through fallopian tubes into peritoneal cavity during menstrual cycle, which disseminates the fragments of endometrial tissues outside the uterine cavity.^13^ Studies published in 1980s have already confirmed that, through laparoscopy conducted during the perimenstrual phase to observe blood in the peritoneal fluid, up to 90% of reproductive‐age women experienced retrograde spillage of menstrual blood.^14^ However, only 10%–15% of them further develop into endometriosis.^14^ These findings indicate that there are additional unknown mechanisms play parts in the pathogenesis of endometriosis.

The subtypes of endometriosis are classified by their phenotypic differences and individual characteristics. First, the superficial endometriosis, also known as the peritoneal endometriosis, is the most common and the mildest one in the subtype. Second, the ovarian endometrioma, so‐called chocolate cyst, is well‐known as its unique cystic morphology on ovaries. Lastly, the DE, defined as the penetration of endometriotic lesions deeper than 5 mm, is considered the most severe type of endometriosis because of the high lesion activities and serious inflammatory‐associated pain. Through the discovery of superficial endometriosis, ovarian endometrioma, and DE, it has been proposed that the etiologies of different types of endometriosis may be different.^15^

According to previous research, the red‐colored superficial endometriosis lesions are considered the early‐stage transplantations from the endometrium through retrograde menstruation, as proposed by Sampson, following by partial shedding, inflammation‐induced scarification, and fibrosis.^16^ These processes lead the lesions to transit from red to black and eventually to white, indicating the quiescent state.^16^ On the other hand, the origin of ovarian endometrioma has been controversial for decades. In 1958, Hughesdon^17^ demonstrated that ovarian endometrioma is caused by cortex invagination due to the accumulation of menstrual debris from the shedding of endometrium. However, the later published research suggested that ovarian endometrioma is originated from the celomic epithelial invagination with metaplastic histogenesis based on their histological observation of the mesothelial invaginated continuum with endometriotic lesions.^18^ As for DE, it has been proposed to be the consequence of mesodermal Müllerian rests and characterized with poor differentiation and hormonal independence based on the immunocytochemical results.^19^ These studies offer a new perspective, suggesting that superficial endometriosis, ovarian endometrioma, and DE may be regarded as distinct entities with their unique pathogenic mechanisms.

## 2 CURRENT TREATMENT OF ENDOMETRIOSIS

The current treatment of endometriosis includes surgical removal and medical treatments (Figure 1), which aim to ease the symptoms caused by the endometriotic lesions. Surgically, multiple guidelines suggested a minimally invasive approach over laparotomy surgery due to its benefits in postoperative pain, duration of hospitalization, recovery, and cosmetic results.^20^ However, the surgical removal of endometrioma significantly impacts on the remaining antral follicular count and anti‐Müllerian hormone.^21^ Moreover, in the comparative study of progestin treatment and surgical interventions, Vercellini et al.^22^, ^23^ demonstrated that treatment with progestin results in the better long‐term symptom relief and patients' satisfactory (59% vs. 43%). Therefore, the consideration of surgical intervention for endometriosis should be carefully evaluated.

Hormonal therapies are commonly used as a symptom‐oriented approach in medical treatment. Androgenic substances were the first to be discovered and used for treating endometriosis.^24^ However, severe side effects promoted the search for more effective and tolerable treatments. Later, several hormonal medications suppressing hypothalamic‐pituitary‐ovarian (HPO) axis were gradually developed, such as Danazol, Gestrinone, combined oral contraceptive pills (COCs), gonadotropin releasing hormones (GnRH) agonists, GnRH antagonists, and multiple progestins (Figure 1). While hormone therapies aim to suppress ovarian activity, progestins act directly on progesterone receptors in endometriotic lesions, which results in the reduction of endometriotic cell proliferation.^25^ To date, COCs and progestins are the most commonly applied hormone therapies for the treatment of endometriosis.

COCs represented a wide variety of estrogen and progesterone combinations. In 2015, it was reported that continuously taking COCs for 6 cycles of medication without surgery significantly decreases endometrioma diameter and improves dysmenorrhea.^26^ On the other hand, ethinylestradiol and drospirenone showed well improvement of pain score and gynecologic symptoms; however, there were still 14.4%–18.3% of the patients remained moderate to severe pelvic discomfort after taking the medications for 24 months.^27^ In addition, for the colorectal endometriosis patients, 30% of them did not significantly improve the symptoms after taking low‐dose estrogen–progestogen combined medications for 12 months, and 16% of them were dissatisfied with the treatment.^28^ These clinical reports demonstrated that while COCs are considered effective as the treatment for endometriosis, parts of the patients still do not benefit from this therapeutic approach.

Addressing endometriosis treatment, progestin‐only treatment is another commonly used therapy often compared to the COCs. In a randomized control trial, the effects of norethindrone acetate were compared to the combined treatment of ethynyl estradiol and cyproterone acetate, which showed the substantially improvement in dysmenorrhea, deep dyspareunia, nonmenstrual pelvic pain, and dyschezia over a 12‐month period.^29^ After decades, the study of dienogest, a newly approved progestin based drug, demonstrated that there is a better improvement of visual analogue scale (VAS) score and endometriosis‐associated pelvic pain (EAPP) after 24 weeks of medications compared to the combined therapy of ethinyl estradiol and drospirenone.^30^ As the evidence indicated that progestin‐only therapy is effective for treating endometriosis, dienogest may be considered the first‐choice medical alternative based on its lower side‐effects, higher tolerability, and better safety.^30^

## 3 PROGESTERONE RESISTANCE

### 3.1 Progesterone resistance in endometriosis

Progesterone resistance is defined as the diminished response while cells are exposed to the progesterone. In endometriosis, attenuation of progesterone response enables the endometriotic cells to continuously grow and survive after translocating to the foreign environment. In the mid‐1980s, Vierikko et al.^31^ noticed that the therapeutic effects of progesterone and progestin in endometriosis patients did not meet the expectations. This research was the first to postulate the development of progesterone resistance during pathogenesis of endometriosis. Decades later, Attia et al.^32^ introduced the concept of progesterone resistance in endometriosis and provided evidences to prove it for the first time.

According to previous studies, hormonal treatment is effective for approximately two‐third of women who suffered from endometriosis‐related pain.^33^ In other words, one‐third of the endometriosis patients are resistant to the current hormone‐based drugs. Several cohort studies have demonstrated that there is a limited long‐term efficacy of progestin treatments.^34^ On the other hand, Ferrero et al.^35^ reported that 6 months of norethindrone acetate treatment resulted in rebound dysmenorrhea and deep dyspareunia. These real‐word investigations indicate the development of progesterone resistance during endometriosis hormonal treatment, which is considered a crucial issue and remained to be solved in the future.

It is well‐known that progesterone works via binding to its specific cognate receptors that belong to the nuclear hormone receptor family. Progesterone receptor (PR) has been identified to have isoform A and B (PR‐A/B) as early as in 1990.^36^ While PR‐A expresses as an 81 kDa protein, PR‐B protein expresses a larger size of 115 kDa because of the additional 164 amino acids at the N‐terminus.^37^ Although PR‐A and PR‐B are nearly identical except the N‐terminal region, more and more evidence showed that these two isoforms are functionally different and mediate the distinct responsive genes which result in specific physiological effects.^38^ The previous studies have demonstrated that while PR‐B acts as the activator to transcribe the progesterone downstream target genes, PR‐A plays the inhibitory role by dominantly repressing PR‐B functions.^39^ Therefore, it is important to have heterogenic expressing ratio of PR‐A to PR‐B in reproductive system to process the progesterone functions in the physiological point of view.^40^

**FIGURE 2:**
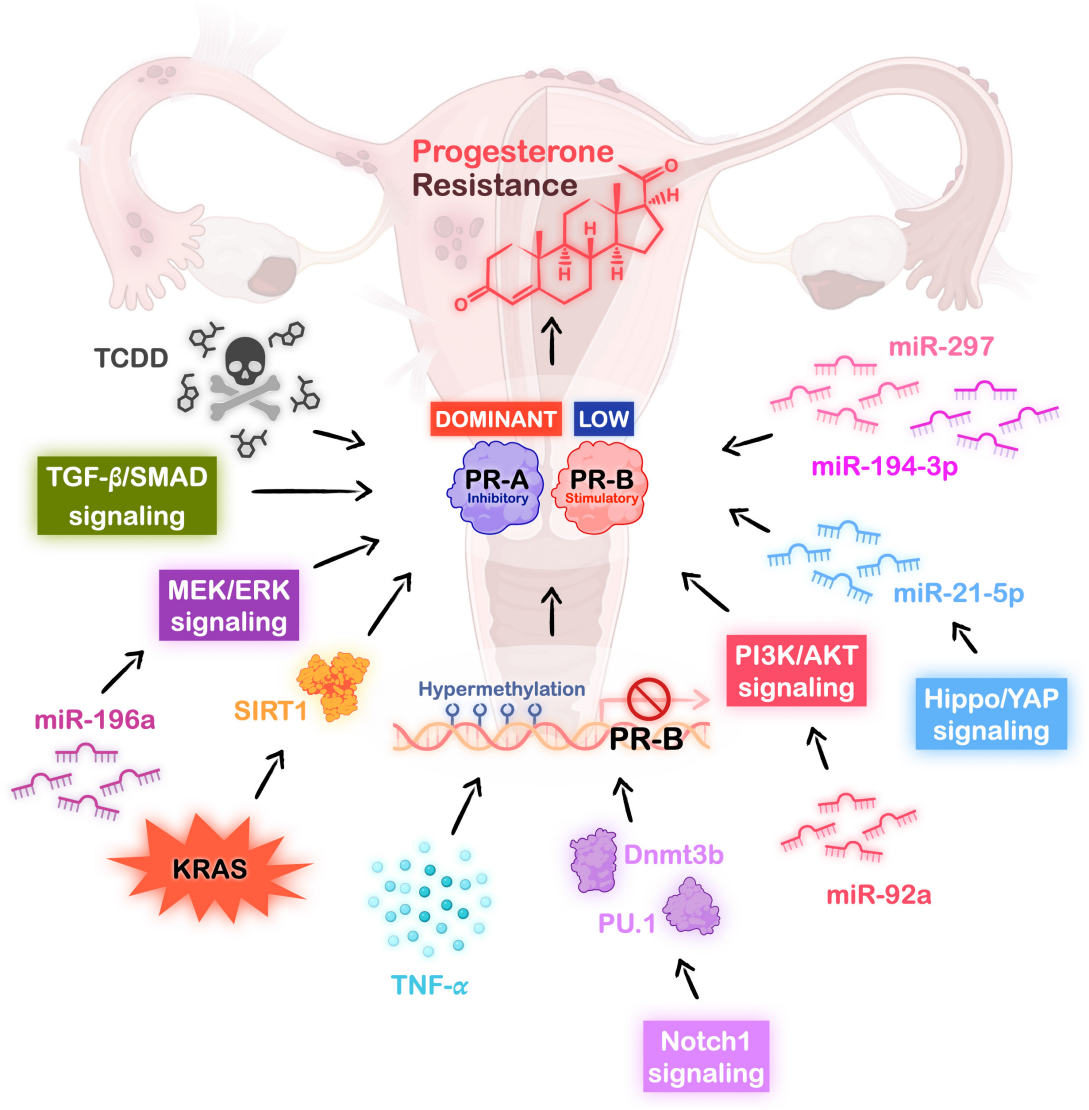
**Potential mechanisms of causing progesterone resistance in endometriosis.** Progesterone resistance in endometriosis has been reported to be associated with exposure of environmental toxin, upregulation of multiple miRNAs, overactive cell signaling pathways, overexpressed KRAS mutation and cytokine effects under chronic inflammation. These regulated pathways eventually result in downregulation of the stimulatory progesterone receptor‐B expression, which further contributes to the development of progesterone resistance in endometriosis. (Dnmt3b, DNA methyltransferase 3b; MEK/ERK, mitogen‐activated protein kinase kinase/extracellular signal‐regulated kinase; miR, microRNA; PI3K/AKT, phosphatidylinositol 3 kinase/protein kinase B; PR, progesterone receptor; SIRT1, Sirtuin 1; TCDD, 2,3,7,8‐tetrachlorodibenzo‐*p*‐dioxin; TGF‐β, transforming growth factor‐beta; TNF‐α, tumor necrosis factor‐α; YAP, yes‐associated protein).

Up to date, PR‐A expression has been found to be predominantly expressed in both eutopic endometrium and endometriotic lesions, especially in the ovarian endometrioma when compared to peritoneal endometriosis.^41^ On the other hand, the downregulation of PR‐B in endometriosis has been linked to the hypermethylation of PR‐B promotor^42^ or by 2,3,7,8‐tetrachlorodibenzo‐*p*‐dioxin, a widespread environmental toxin.^43^ These data indicate that epigenetic regulation or microenvironmental pollutants may promote progesterone resistance in endometriosis (Figure 2).

### 3.2 Novel hormonal therapy aiming to overcome progesterone resistance

In addition to modifying progestin side chain to overcome receptor modulation in progesterone resistance, ongoing research focuses on the development of various hormonal suppressive medications that target distinct pathways. The recently discovered novel medicines include selective estrogen receptor modulators (SERMs), selective progesterone receptor modulators (SPRMs), and oral GnRH antagonists (Figure 1).

SERMs are ligands for estrogen receptors, exhibiting either agonistic or antagonistic effects when target specific tissues. SERMs were derived from the medications that were designed for preventing the osteoporosis; these drugs characterize the estrogenic effects in bone while having anti‐estrogenic effects in endometrium.^44^ Raloxifene is the most well‐defined SERMs, which displayed the effects of lesion regression in animal models.^45^ Except for raloxifene, TAS‐108 is another well‐known SERMs, which showed strong inhibitory effects on the transplanted endometrial cysts in animal models.^46^ Besides, the other SERM, SR‐16234, was reported to significantly reduce pelvic pain, dysmenorrhea, stiffness of Douglas' pouch, and limitations in uterine movement after 12 weeks of treatment.^47^ However, Stratton et al. showed that raloxifene treatment shortened the relapse time of endometriosis‐induced chronic pelvic pain in a randomized control trial.^48^ Therefore, further studies of SERMs are required to better evaluate the feasibility of these drugs for endometriosis treatment.

As for SPRMs, they are progesterone receptor ligands exhibiting either agonistic or antagonistic effects when targeting distinct tissues.^49^ SPRMs were reported to inhibit ovulation, suppress endometrial cell proliferation, ease endometrial bleeding, and relieve the endometriosis‐associated pain.^50^ Regarding SPRMs, RU486 (Mifepristone) is the most studied one in endometriosis. According to Kettel et al.,^51^ 6 months of RU486 low‐dose treatment simultaneously induced the ovarian acyclicity and improved the EAPP. A decade later, in an open‐label phase II/III trial (NCT02271958), the treatment of endometriosis patients with 2.5, 5, and 10 mg of RU486 resulted in the reduction of dysmenorrhea to only 10.2%, 1.1%, and 1.1%, respectively. However, this drug still caused adverse effects for endometriosis patients such as hot flush (15.1%), nausea (2.3%), vomiting (1.2%), elevated liver enzyme (3.4%), endometrial proliferation (34.7%), and progesterone associated endometrial changes (16.7%).^52^, ^53^ To overcome the side effects, a series of new SPRMs were developed including asoprisnil, telapristone acetate, ulipristal acetate, and vilaprisan.^54^ A recent phase I/II trial revealed that vilaprisan was well‐tolerated, exerts minimal hepatic toxicity, and has a favorable pharmacokinetic profile.^55^ Larger cohort studies and clinical trials are still ongoing to examine the efficacy and safety of vilaprisan, with results anticipated to be reported soon.

GnRH antagonists have been well‐developed for endometriosis treatment, with oral medications emerging in recent years. In contrast to injectable GnRH agonists, oral GnRH antagonists inhibit the secretion of estrogen in a dose‐dependent manner without causing flare‐up effects, the reversal of estrogen‐suppression effects is rapid and can be achieved shortly after discontinuation of the drugs.^56^ Moreover, in a phase II trial, a GnRH antagonist, linzagolix exhibited potential efficacy for EAPP, and their effectiveness is currently being evaluated in ongoing phase III trials.^57^ Further studies are necessary to compare the efficacy and safety between GnRH antagonist and current therapeutic options with a long‐term follow‐up to enhance our understanding of this new class of drugs.

Although the dienogest treatment has been well established and responded, there are still exceptions. In an open label trial, it was reported that about 30% of endometriosis patients who received dienogest treatment for 28 weeks did not improve the EAPP.^58^ Besides, a large prospective cohort study with participation of 3113 endometriosis patients showed that 23% of them had no obvious effects after dienogest treatment.^59^ In 2022, a real‐world clinical practice, including endometriosis patients from 6 Asian countries, revealed that 2 mg dienogest treatment for 24 months did not improve the EAPP in 16.3% of the patients while 5.1% patients among them were even deteriorated.^60^ This study also uncovered that although treating with dienogest in the first 6 months ameliorated the EAPP, the results yet deteriorated after 24 months of the medication.^60^ Based on these clinical studies, we could assume that under long‐term dienogest treatment, parts of the endometriosis patients may develop drug resistance.

## 4 PROGESTERONE RESISTANCE REGULATED BY CELL SIGNALING IN ENDOMETRIOSIS

Despite the existence of innovative therapeutic approaches based on the selective PR targeting characteristics to overcome progesterone resistance, it is important to study the molecular mechanisms of progesterone resistance to discover additional novel therapeutics targets because roughly one‐fifth of the patients are still not responding to progesterone treatment. So far, most of the studies reveal that downregulation of PR, especially the functional PR‐B is the key component of developing progesterone resistance; therefore, understanding the mechanisms responsible for PR‐B downregulation in endometriotic lesion is a key to overcome this obstacle. Besides affected by the epigenetic regulation and environmental toxin, the dysregulation of cell signaling in endometriosis also plays a role during the development of progesterone resistance, which are summarized below.

### 4.1 The HIPPO pathway

The Hippo pathway is a conserved signaling pathway that plays a crucial role in processes such as organ development, tissue regeneration, and immune modulation.^61^ The core components of Hippo pathway include the kinase cascade MST1/2 and LATS1/2 along with the downstream effectors, Yes‐associated protein 1 (YAP1), and transcriptional coactivator with PDZ‐binding motif (TAZ), which later contribute to the transcriptional regulation leading to various cellular responses.^61^ It has been reported that YAP1 activity is increased in endometriotic lesion via hypoxia‐suppressed LAST1‐mediated YAP1 phosphorylation^62^ or through anthrax toxin receptor 2‐induced YAP1 nuclear translocation.^63^ Interestingly, upregulation/activation of YAP1 resulted in PR downregulation through microRNA (miR)‐21‐5p‐mediated mRNA degradation,^64^ which not only causes the progesterone resistance but also leads to decidualization impairment (Figure 2). In contrast, PR expression can be rescued by blocking YAP1 signaling, either through knockdown of YAP1 in the human ectopic endometrial stromal cells or administration of the YAP1 inhibitor, verteporfin.^64^ More intriguingly, the use of verteporfin in the endometriosis mouse model showed that targeting YAP1 signaling effectively attenuates the development of endometriotic lesions without reducing reproductive ability and the growth of offspring.^62^ These data suggest that targeting the YAP1 signaling pathway can reduce progesterone resistance without compromising the reproductive capacity of the mother or causing adverse effects on offspring.

### 4.2 MicroRNA and ERK signaling pathway

MicroRNAs are the small noncoding RNAs with gene‐regulated functions by inducing mRNA degradation or repressing translational process. Studies revealed that miRNAs play a crucial part in pathogenesis of endometriosis, including causing progesterone resistance.^65^ For example, it has been shown that miRNAs like miR‐194‐3p and miR‐297 act to inhibit the PR expression in endometriosis which leads to defective decidualization and progesterone resistance^66^, ^67^ (Figure 2). Another study also showed that miRNA‐196a regulates PR level through mediating mitogen‐activated protein kinase (MAPK) kinase/extracellular signal‐regulated kinase (ERK) signaling activation in endometriosis^68^ (Figure 2). In fact, MAPK signaling activation has been reported to downregulate PR‐B expression by a ligand‐dependent process in a breast cancer study.^69^ Later research also confirmed that MEK/ERK signaling is highly activated in endometriosis, which results in aberrant expression of PR.^70^ Fortunately, inhibition of MEK1/2 by using U0126 inhibitor successfully increases the PR levels, including total PR and nuclear PR‐A/B protein expressions in ectopic endometrial stromal cells.^70^ However, in normal human eutopic endometrial stromal cells, the human chorionic gonadotropin (hCG)‐induced ERK1/2 signaling activation was reported to increase the PR expression, and the effect could be abolished by treating with U0126.^71^ These studies reveal that even the identical cell signaling activation, in response to different stimuli and in normal endometrium or endometriotic lesions, leads to completely divergent regulation of PR expression.

### 4.3 The PI3K/AKT signaling pathway

The phosphatidylinositol 3 kinase/protein kinase B (PI3K/AKT) signaling was also found to be highly activated in ovarian endometrioma and DIE while compared to normal endometrium.^72^ The activated PI3K/AKT signaling has been identified to promote the endometriotic lesion progression, reduce fertility through attenuating decidualization and decreasing ovarian reservation, and contribute to the progesterone resistance.^73^, ^74^, ^75^ Previous studies have demonstrated that PR levels are downregulated under overactivation of PI3K/AKT signaling in endometriosis.^70^, ^74^ Nonetheless, by knocking down AKT or treating with AKT inhibitor, MK‐2206, to block PI3K/AKT signaling in primary cultured endometriotic stromal cells, the total and nuclear PR‐A/B expressions can be restored again.^70^ Moreover, Li et al.^76^ discovered that the upregulation of miR‐92a in endometriosis further leads to progesterone resistance by suppressing the level of phosphatase and tensin homolog (PTEN), a negative regulator of AKT signaling (Figure 2). On the contrary, inhibiting miR‐92a with its antagomir enhanced the progesterone therapeutic effects by reducing the endometriotic stromal cell proliferation, which in turn decreases the number and size of endometriotic lesions in the endometriosis mouse model.^76^

### 4.4 The Notch signaling pathway

According to previous studies, several lines of evidence indicated that Notch1 signaling is highly activated in endometriotic lesions,^77^ which functions to promote the cell proliferation, cell invasion, and angiogenesis during the pathogenesis of endometriosis.^78^, ^79^ In 2016, Su et al.^80^ discovered that Notch1 signaling hyperactivation contributes to PR loss through hypermethylation of PU.1 and DNA methyltransferase 3b (Dnmt3b)‐mediated transcription factor, which not only results in dysregulation of progesterone and estrogen signaling but also contributes to complete infertility (Figure 2). A later published research also confirmed that highly activated Notch1 signaling leads to the progesterone resistance through lower the transcripts of total PR and PR‐B in ectopic endometrial tissues.^81^ Conversely, inhibition of Notch1 signaling by using DAPT, a 𝛾‐secretase inhibitor, which prevents the cleavage process in releasing the bioactive notch intracellular domain (NICD) of the Notch1 receptor, successfully enhanced progesterone sensitivity by increasing PR transcript levels and PR nuclear protein expression in human endometrial stromal cells.^81^

### 4.5 Other signaling pathways

Except for the cell signaling pathways mentioned above that play parts in the progesterone resistance in endometriosis, it was reported that the activating mutation of KRAS in endometriosis upregulates Sirtuin 1 (SIRT1), which results in compromising progesterone responses^82^ (Figure 2). Another study also pointed out the importance of overexpressed SIRT1 in endometriosis, which not only contributes to progesterone resistance but also impacts on decidualization and implantation processes.^83^ Interestingly, the administration with SIRT1 inhibitor, EX‐527, ameliorated the implantation failure and attenuated the development of endometriotic lesions in the endometriosis mouse model.^83^ Moreover, a recent published article demonstrated that the well‐known cytokine‐mediated cell signaling, transforming growth factor‐beta (TGF‐β)/SMAD signaling, reduces the PR‐B protein expression and the endometrial receptivity in primary cultured eutopic endometrial stromal cells, potentially leading to infertility in women with endometriosis^84^ (Figure 2).

## 5 POTENTIAL THERAPEUTIC STRATEGIES TO OVERCOME PROGESTERONE RESISTANCE

**FIGURE 3:**
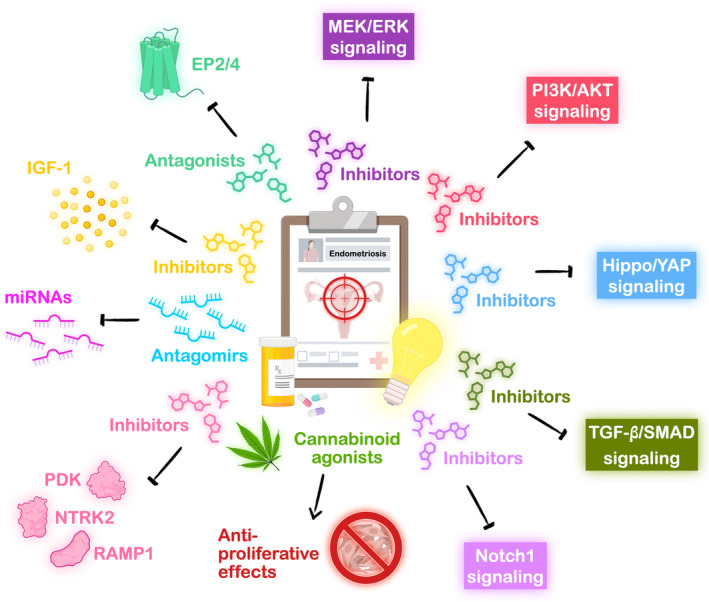
**Potential therapeutic targets for endometriosis treatment.** Summary of potential therapeutic approaches outlined in the published literature in the studies of endometriosis. The strategies include blocking the overactive cell signaling pathways through the use of inhibitors, antagonizing certain highly expressed miRNAs with antagomirs, repressing specific aberrantly expressed receptors or enzymes with antagonists or inhibitors, and adopting the antiproliferative properties of cannabinoids. These therapeutic targets collectively aim to alleviate the pathological progressions of endometriosis and concomitantly ease the endometriosis‐associated pain symptoms (EP2/4, prostaglandin E_2_ receptors 2/4; IGF‐1, insulin‐like growth factor‐1; MEK/ERK, mitogen‐activated protein kinase kinase/extracellular signal‐regulated kinase; miR, microRNA; NTRK2, neurotrophic receptor tyrosine kinase 2; PDK, pyruvate dehydrogenase kinase; PI3K/AKT, phosphatidylinositol 3 kinase/protein kinase B; PR, progesterone receptor; RAMP1, receptor activity‐modifying protein 1; SIRT1, Sirtuin 1; TCDD, 2,3,7,8‐tetrachlorodibenzo‐*p*‐dioxin; TGF‐β, transforming growth factor‐beta; TNF‐α, tumor necrosis factor‐α; YAP, yes‐associated protein).

Uncovering the molecular mechanisms behind the development of progesterone resistance during endometriosis is considered important at all times. As progesterone resistance cuts down the therapeutic effects in endometriosis patients, it is crucial to search for the potential targets or find the new adjuvants to support the current therapeutic regimens for improving the treatment outcomes of endometriosis. Since miRNAs like miR‐21‐5p, miR‐194‐3p, miR‐297, miR‐196a, and miR‐92a have been identified to regulate progesterone resistance by inhibiting the PR expression in endometriotic lesions,^64^, ^66^, ^67^, ^68^, ^76^ employing the certain miRNA antagomirs may be the possible strategy for the therapeutic approach of endometriosis (Figure 3). Additionally, the utilization of specific inhibitors to target the cell signaling pathways associated with progesterone resistance in endometriosis, such as Hippo/YAP signaling, MEK/ERK signaling, PI3K/AKT signaling, Notch1 signaling, and TGF‐β/SMAD signaling,^64^, ^70^, ^81^ may offer potential opportunities for developing effective adjuvants in clinical application (Figure 3).

Besides discovering potential targets aimed at overcoming progesterone resistance in endometriosis, it is essential to identify the therapeutic targets based on the pathological molecular mechanisms that disrupt the development of endometriosis and alleviate the troublesome symptoms such as the serious neuropathic pain induced by the chronic inflammation. For example, prostaglandin E_2_ (PGE_2_) has been shown to regulate endometriotic lesion development, angiogenesis, and immune privilege^85^, ^86^, ^87^, ^88^, ^89^, ^90^, ^91^; therefore, it is reasonable to hypothesize that inhibiting PGE_2_ signaling may ameliorate disease burden. Indeed, Arosh et al.^92^ revealed that suppressing PGE_2_ receptors, EP2 and EP4, by using combined treatment of the inhibitors, AH6809 and AH23848, successfully reduces the endometriotic lesions and eases the pain responses in the endometriosis mouse model (Figure 3). Subsequent research showed that treatment with EP2 antagonist, PF04418948, in endometriosis mouse model efficiently improved the hyperalgesia of neuroaxis to lower the endometriosis‐associated pain.^93^ Other example such as administration of insulin‐like growth factor‐1 (IGF‐1) inhibitor, linsitinib, in endometriosis mouse model reversed the pain behavior by decreasing neuronal growth^94^ (Figure 3). Moreover, it has been reported that targeting the pyruvate dehydrogenase kinase/pyruvate dehydrogenase, neurotrophic receptor tyrosine kinase 2, calcitonin gene‐related peptide receptor/receptor activity‐modifying protein 1, or VEGF‐C in endometriosis mouse model effectively inhibits the endometriotic lesion growth either through abrogating cell proliferation, promoting cell apoptosis, or suppressing angiogenesis/lymphangiogenesis^5^, ^95^, ^96^ (Figure 3).

Interestingly, cannabinoids, the natural chemical compounds found in the Cannabis plant, have been identified to have antiproliferative effects through inhibiting growth factors and downregulating the signaling pathways like MEK/ERK signaling and PI3K/AKT signaling.^97^, ^98^ In 2010, Leconte et al. revealed that cannabinoid agonist, WIN55212‐2, carried out the antiproliferative effect by inhibiting AKT signaling in primary cultured endometriotic stromal cells isolated from DIE nodules^99^ (Figure 3). Moreover, in the DIE nodules‐grafted mice model, the application of WIN55212‐2 showed a reduction in the volume of DIE implants, which confirms the beneficial effects of cannabinoid agonist.^99^ A decade later, another research team suggested that treatment with the psychoactive constituent of Cannabis plant, Δ9‐tetrahydrocannabinal, not only relieved the endometriosis related pain and the anxiety behaviors but also alleviated the endometriotic cysts formation in the endometriosis mouse model.^100^

These studies demonstrated that targeting aberrantly regulated receptors/enzymes or utilizing natural cannabinoids yields positive improvements in the outcomes of endometriosis pathogenesis. This concept may provide a novel perspective as a potential nonhormonal therapeutic strategy for endometriosis in the future.

## 6 CONCLUSION REMARKS

Endometriosis has been considered as a complex disorder regarding the distinct etiologies among its subtypes, a notable recurrence rate following the surgical intervention, and the treatment challenges attributed to progesterone resistance. Though the collaborative efforts of researchers and clinicians, new drugs for endometriosis, like SERMs and SPRMs, have been discovered to overcome the challenges encountered in the treatment of patients with endometriosis. However, there are still exceptions that do not adapt to these newly discovered drugs.

Fortunately, researchers have dug into the molecular mechanisms of endometriosis, and revealed the crucial roles of several signaling pathways such as Hippo signaling pathway, ERK signaling pathway, PI3K/AKT signaling pathway, and Notch signaling pathway in the pathogenesis of endometriosis as well as in the regulation of progesterone resistance. We believe that developing the novel drugs through targeting or blocking specific signaling pathways may be the promising therapeutic strategy for endometriosis treatment in the future. In addition, developing new therapeutic approaches based on the recent insights gained from the studies of miRNA may also offer a hope for the treatment of endometriosis.

## CONFLICT OF INTEREST STATEMENT

We have no known conflict of interest to disclose.
